# Short‐term benefits of image‐based robotic assistance in TKA after prior HTO: A comparative study

**DOI:** 10.1002/jeo2.70757

**Published:** 2026-06-11

**Authors:** Sarah Descombris, Guillaume Mesnard, Hannes Vermue, Cécile Batailler, Elvire Servien, Sébastien Lustig

**Affiliations:** ^1^ Orthopaedics Surgery and Sports Medicine Department, FIFA Medical Center of Excellence, Croix‐Rousse Hospital, Hospices Civils de Lyon Lyon North University Hospital Lyon France; ^2^ Department of Orthopaedic Surgery and Traumatology Ghent University Hospital Ghent Belgium; ^3^ Univ Lyon Claude Bernard Lyon 1 University Lyon France; ^4^ LIBM—EA 7424, Interuniversity Laboratory of Biology of Mobility Claude Bernard Lyon 1 University Lyon France

**Keywords:** femorotibial osteoarthritis, high tibial osteotomy, robotic‐assisted total knee arthroplasty, total knee arthroplasty

## Abstract

**Purpose:**

High tibial osteotomy (HTO) is used to treat medial knee osteoarthritis with varus alignment in young, active patients. While robotic‐assisted total knee arthroplasty (TKA) may improve implant positioning and soft‐tissue balance, its benefits in post‐HTO patients remain unclear. This study compares surgical, clinical and radiological outcomes between robotic‐assisted and conventional TKA following HTO.

**Methods:**

A retrospective study included 87 patients with prior HTO who underwent TKA between August 2017 and October 2023 (46 robotic‐assisted and 41 conventional), with a minimum 1‐year follow‐up. Demographic data, surgical characteristics, clinical outcomes (Knee Society Score [KSS], range of motion), radiological alignment (hip–knee–ankle angle) and complications were analysed. Mean follow‐up was 23 months.

**Results:**

Operative time was similar between groups (82 vs. 95 min; *p* = 0.07). Robotic‐assisted TKA was associated with fewer lateral releases (30% vs. 54%; *p* = 0.03) and no tibial tubercle osteotomies (0% vs. 10%; *p* = 0.03). Cruciate‐substituting implants were used more frequently in the robotic group (13% vs. 0%; *p* = 0.001), whereas stemmed implants were more common in the conventional group (49% vs. 17%; *p* = 0.001). Postoperative KSS Knee scores were comparable between groups (*p* = 0.28), while KSS Function scores were slightly higher in the conventional group (*p* = 0.048). Complication rates did not differ significantly.

**Conclusion:**

Robotic‐assisted TKA after HTO was associated with a similar operative times and comparable short‐term clinical outcomes to conventional TKA. It was also associated with lower rates of additional procedures and a less frequent use of stemmed or constrained implants. These findings should be interpreted with caution, given the baseline differences between the groups. Longer follow‐up is warranted.

**Level of Evidence:**

Level III, therapeutic studies, retrospective comparative study.

**Abbreviations**
ASA: American Society of Anaesthesiologists
BMI: body mass index
CCK: constrained condylar knee
CS: cruciate substituting
CT: computed tomography
DAIR: Debridement, Antibiotics and Implant Retention
HKA: hip–knee–ankle angle
HTO: high tibial osteotomy
KSS: Knee Society Score
LCW: lateral closing wedge
MCID: minimal clinically important difference
mLDFA: mechanical lateral distal femoral angle
MOW: medial opening wedge
MP: medial parapatellar
MPTA: medial proximal tibial angle
PJI: periprosthetic joint infection
PS: posterior stabilized
ROM: range of motion
SV: subvastus
TKA: total knee arthroplasty

## BACKGROUND

High tibial osteotomy (HTO) is a well‐established technique for the treatment of low‐grade medial osteoarthritis of the knee with varus alignment, especially in young and active patients [25]. Although the long‐term survival of HTO has improved over recent years [16], it nonetheless remains limited, with reported rates of only 63% at 15 years [2, 3]. In most cases, disease progression necessitates conversion to total knee arthroplasty (TKA) after several years.

Conversions to TKA are more technically demanding than TKAs performed on knees that have not undergone previous surgery. This is due to various surgical challenges, such as a more difficult approach, ligamentous imbalance and anatomical distortion of the proximal tibial metaphysis resulting from previous osteotomies [7, 19]. In fact, several studies have reported longer operating times and greater blood loss in cases of TKA where HTO had not previously been performed [7, 19]. These factors are largely attributed to more difficult joint exposure, often requiring additional procedures such as tibial tubercle osteotomy, lateral retinacular release or medial/lateral soft tissue release [19]. While the functional results appear comparable between post‐HTO TKA and TKA without a previous HTO [20, 22]. The complexity of the procedure contributes to a higher revision rate in post‐HTO TKA (7.6% vs. 3.7%) [7]. The leading causes of revision are aseptic implant loosening and deep infection [7], with the increased infection rate potentially linked to prolonged operative time and history of internal fixation [22].

Given these challenges, robotic‐assisted surgery may offer advantages in these complex cases. Recent meta‐analyses have demonstrated that robotic assistance enables more precise implant position in the frontal, sagittal and axial planes, while accounting for soft‐tissue laxity, compared to conventional TKA [14, 27].

However, no studies have directly compared the surgical, clinical and radiological outcomes of conventional TKA versus robotic‐assisted TKA following HTO. The aim of this study was therefore to assess the benefits of image‐based robotic assistance for TKA after HTO. Firstly, we investigated whether operative times differed between robotic‐assisted and conventional TKAs in patients with a history of HTO. We hypothesize that TKAs performed with robotic assistance do not have a longer operative time than those performed with conventional instrumentation. Secondly, we evaluated various surgical and clinical outcomes, including exposure difficulties and the level of implant constraint, as well as clinical outcomes such as range of motion (ROM), early complications at 2 months and functional scores at the final follow‐up. Finally, we assessed whether there were differences in radiological outcomes, such as component alignment and lower limb mechanical axis, between robotic‐assisted and conventional TKAs in patients who had undergone HTO.

## MATERIALS AND METHODS

**Figure 1:**
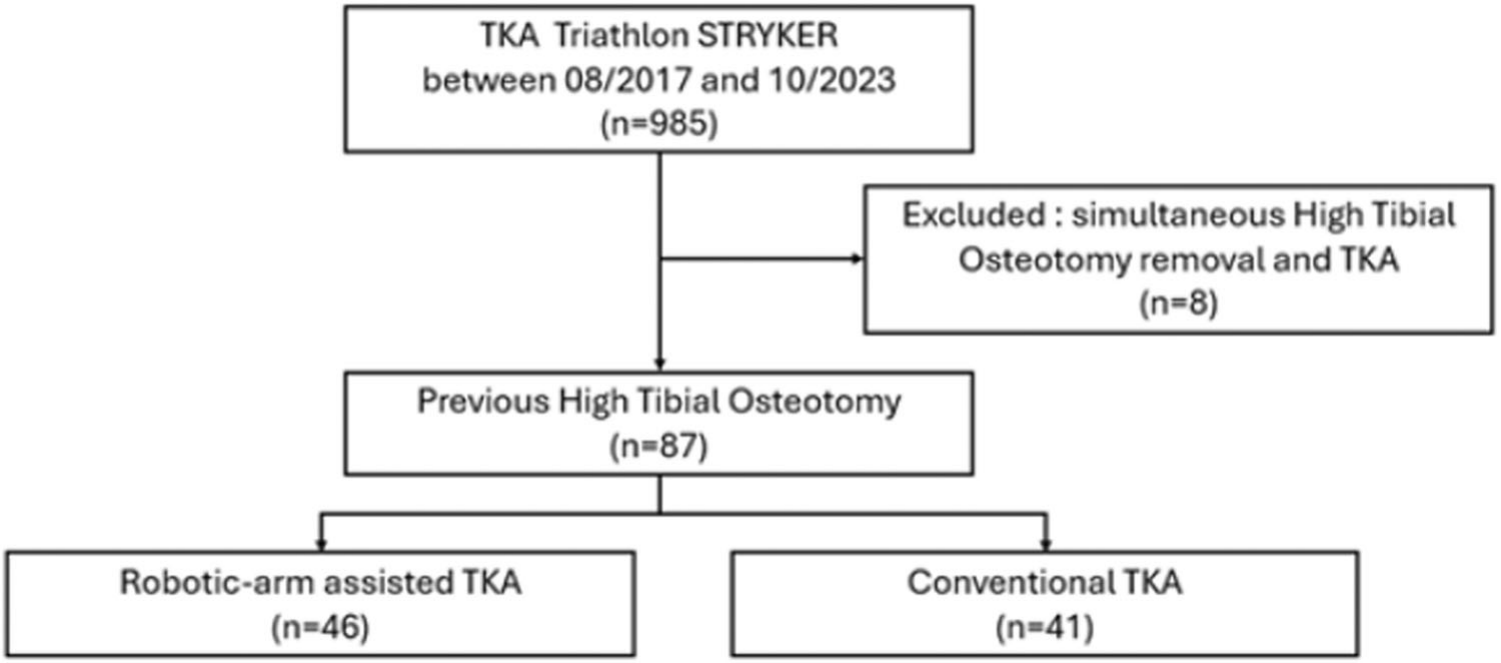
Flow chart. TKA, total knee arthroplasty.

A retrospective study based on prospectively collected data was performed in a tertiary referral centre specializing in primary and revision TKA. Between August 2017 and October 2023, a total of 985 TKAs using the Triathlon implant (Stryker) were performed. Patients with a history of HTO prior to TKA were identified through a review of medical records. The indication for HTO was symptomatic medial tibiofemoral osteoarthritis in young, active patients with varus alignment. Patients with a history of infection, ligamentous insufficiency, femoral or tibial fractures, or concomitant HTO with TKA due to malalignment were excluded. Of the eligible patients, 87 were found to have undergone TKA with a history of HTO. Of these, 46 underwent robotic‐arm‐assisted surgery and 41 were treated with conventional ancillary (Figure 1). All procedures were performed by two experienced arthroplasty surgeons. All procedures were performed in accordance with the ethical standards of the institutional and/or national research committee, the 1964 Helsinki declaration and its later amendments or comparable ethical standards. Data collection and analysis were carried out in accordance with MR004 Reference Methodology from the Commission Nationale de l′Informatique et des Libertés (Ref. 2238218v0) obtained on 24 March 2025. The study was registered and filed on the Health Data Hub website.

### Patient demographics

The patients' age at the time of both TKA and the HTO, age, body mass index (BMI), preoperative ROM and preoperative KSS (Knee Society Score) were similar between the two groups (Table 1) [8]. However, the American Society of Anaesthesiologists (ASA) score was significantly higher in the conventional TKA group (2.1 range [1–3] vs. 1.7, range [1–4], *p* = 0.006).

**Table 1:** Preoperative clinical and radiological data.

Variable	Robot‐assisted TKA (*n* = 46)			Conventional TKA (*n* = 41)			*p* Value*
	*N*	Mean ± SD	(Min–Max)	*N*	Mean ± SD	(Min–Max)	
Age at the time of HTO (years)		48 ± 10	(16–69)		47 ± 13	(17–66)	0.200
Time from HTO to TKA (years)		13 ± 7	(3–46)		17 ± 9	(1–48)	0.690
Age at the time of TKA (years)		62 ± 10	(38–83)		65 ± 9	(47–86)	0.195
Sex (percent male)	32/46 (69.6%)			25/41 (63.4%)			0.546
ASA Score		2 ± 1	(1–3)		2 ± 1	(1–4)	0.006*
BMI (kg/m²)		28 ± 3	(22–38)		29 ± 5	(19–39)	0.219
Flexion contracture (degrees)		3 ± 4	(0–15)		3 ± 4	(0–10)	0.762
Flexion (degrees)		121 ± 12	(90–140)		116 ± 17	(90–140)	0.113
KSS Knee		66 ± 13	(33–94)		60 ± 16	(22–80)	0.055
KSS Function		71 ± 14	(25–100)		67 ± 16	(15–90)	0.197
MOW HTO	36/46 (79%)			23/41 (56%)			0.027*
LCW HTO	10/46 (21%)			18/41 (44%)			0.027*
Alhbäck Score		3 ± 0.6	(2–4)		4 ± 1	(3–4)	<0.0001*
HKA angle (degrees)		178 ± 4.8	(169–188)		180 ± 7	(168–200)	0.200
mLDFA angle (degrees)		89 ± 2.4	(85–98)		89 ± 3.7	(82–97)	0.482
MPTA angle (degrees)		91 ± 4.1	(83–99)		91 ± 4.7	(80–100)	0.680
Caton‐Deschamps Index		0.9 ± 0.2	(0.4–1.3)		1.0 ± 0.9	(0.4–1.2)	0.293

*Note*: Continuous data are displayed as mean ± SD (range). Categorical data are mentioned in percentages.

Abbreviations: ASA, American Society of Anesthesiologists; BMI, body mass index; HKA, hip–knee–ankle angle; HTO, high tibial osteotomy; KSS, Knee Society Score; LCW, lateral closing wedge; mLDFA, mechanical lateral distal femoral angle; MOW, medial opening wedge; MPTA, medial proximal tibial angle; SD, standard deviation; TKA, total knee arthroplasty.

* Statistically significant with *p* < 0.05.

There were more medial opening wedge HTOs in robotic‐assisted group compared to the conventional group (79% vs. 56%, *p* = 0.027). The Ahlbäck score was significantly higher in the conventional group (3, [range, 2–4] vs. 4, [range, 3–4], *p* < 0.001). Preoperative frontal deformity was comparable between the two groups (Table 1), with an average hip–knee–ankle (HKA) angle of 178.3° (range, 169–188) in the robotic‐assisted group and 179.9° (range, 168–200) in the conventional group (*p *= 0.20). The Caton‐Deschamps ratios were similar in both groups (Table 1).

Follow‐up was 16 months (range, 12–37) in robotic‐assisted group TKA and 30 months (range, 12–62) in the conventional group (*p* < 0.0001).

### Outcome measures

Surgical variables were extracted from the patient's electronic files. These include operative time, surgical approach and related concomitant procedure other than TKA (such as lateral release, posterior capsular release or tibial tubercle osteotomy).

The subvastus (SV) approach was used as the standard exposure in all cases unless preoperative examination or radiographs demonstrated significant lateral patellar maltracking, in which case a medial parapatellar (MP) approach was used. In case lateral patellar maltracking was combined with a history of prior lateral incision or dense lateral scarring, a lateral parapatellar approach was selected. Lateral retinacular release was performed only when patellar tracking remained inadequate following component positioning and trial reduction. Tibial tubercle osteotomy was reserved for cases with severe patella baja, fixed patellar maltracking or inability to achieve safe exposure without excessive tension on the extensor mechanism.

The type of implant, the stems used and the type of implant constraint were noted. The latter could be a cruciate substituting (CS) implant, a posterior stabilized (PS) implant or a constrained condylar knee (CCK) implant. The choice between CS and PS implants was at the operating surgeon's discretion. CCK implants were used for patients presenting with increased laxity on stress radiographs or in cases of increased laxity intraoperatively. Short cemented tibial stems were used when metaphyseal bone stock was compromised or additional stability was deemed necessary based on the surgeon's assessment during the operation.

Both groups underwent clinical and radiological evaluations. Clinical outcomes were assessed based on the ROM at 2 months postoperatively. KSS scores were recorded at the final follow‐up. Pre‐ and postoperative radiographic evaluations included anteroposterior standing long‐leg X‐rays, lateral views and skyline views of the patella at 30° of knee flexion. The Ahlbäck classification was used to assess the level of osteoarthritis [1]. The HKA was measured to assess frontal plane deformity. HKA values below 180° indicate varus alignment, whereas values above 180° indicate valgus alignment. All measurements were performed by one of the authors, who was blinded to the study cohort. Postoperative complications were collected according to the Knee Society at 2 months [12].

### Data analyses

All statistical analyses were performed using XLSTAT® software (Lumivero). Continuous variables were reported as means with range or medians with interquartile ranges, depending on normality, which was assessed using the Shapiro–Wilk test. Categorical variables were presented as frequencies and percentages. Group comparisons were conducted using the Student's *t* test with Welch's correction for continuous variables and the *χ* ^2^ or Fisher's exact test for categorical variables. In case of differences in KSS scores, these were compared to minimal clinically important difference (MCID) values of seven point two and nine point seven for KSS Knee and KSS Function, respectively, to assess whether these were clinically meaningful [15]. An a priori power analysis was performed with G*Power 3.1 (Heinrich‐Heine‐Universität Düsseldorf). For the effect size, a standard deviation of 15 min was assumed based on prior single‐surgeon TKA series, and a clinically relevant between‐group difference of 15 min was selected, given evidence that each additional ten minutes of operative time increases postoperative complication risk [5, 23, 24]. To reach a power of 80%, 37 patients were necessary per cohort, resulting in a total of 74 patients. The significance threshold was set at *p* < 0.05.

## RESULTS

### Surgical approach and exposure

Operative time was comparable between the robotic and conventional group (82 min, range [37–178] vs. 95 min, range [50–160]; *p* = 0.07). All TKAs in the robotic‐assisted group performed with a medial‐sided approach, including 41 SV approaches and 5 MP approaches, according to the operator's preference. In the conventional group, 32 TKAs were performed with the SV approach (*p* = 0.164) and five with the MP approach (*p* = 0.849). Additionally, four TKAs in the conventional group were performed with a lateral parapatellar approach (*p* = 0.03); two of these were due to significant valgus deformity (HKA of 195° and 200°) and two were required due to concurrent lateral HTO material removal performed during TKA.

**Figure 2:**
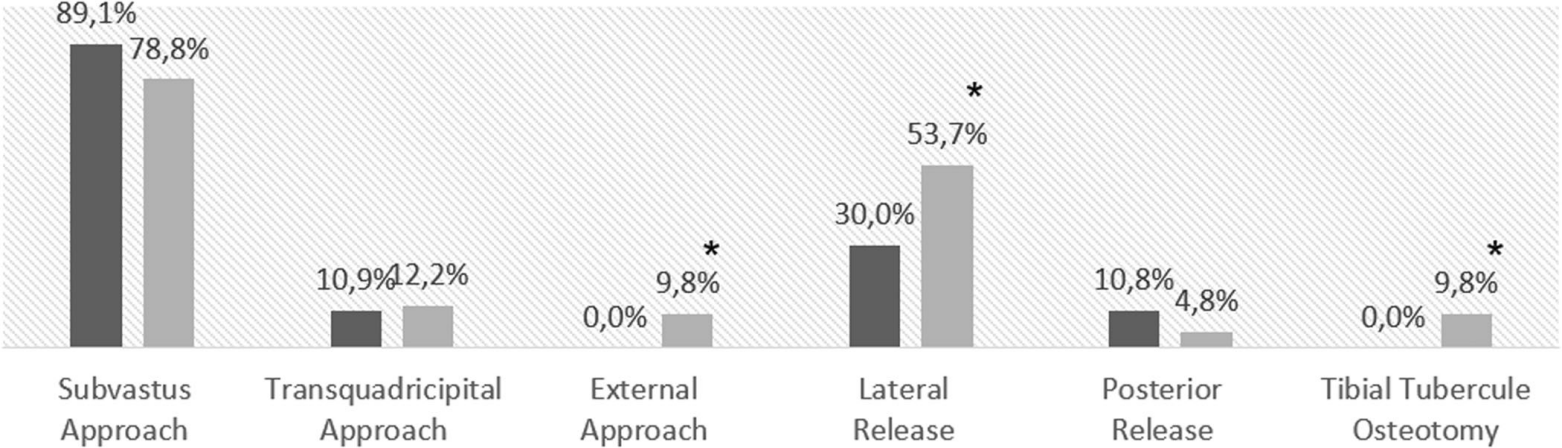
Approach and exposure. *Statistically significant with *p* < 0.05.

No medial releases were performed in either group. The posterior capsular release was similar in both groups (10% in the conventional group and 5% in the robotic group; *p* = 0.31). It was performed due to persistent fixed flexion deformity following the initial bone cuts. The conventional group had a higher incidence of lateral releases (30% vs. 54%, *p* = 0.03) and tibial tubercle osteotomy (0% vs. 10%, *p* = 0.03) (Figure 2).

### Implant constraint level

All TKAs were performed using the Triathlon implant (Stryker Corporation). No CS implants were used in the conventional group, whereas six (13%) were used in the robotic‐assisted group (*p* = 0.001). The conventional group had a higher proportion of PS implants (25 [54%] vs. 32 [78%], *p *= 0.02). The proportion of CCK implants was similar in both groups. Stemmed implants were more frequently used in the conventional group (20 [49%] vs. 8 [17%], *p* = 0.001). There were no significant differences in polyethylene thickness between the two groups (Table 2).

**Table 2:** Overview of the insert thicknesses used in both the robotic‐assisted and conventional TKA cohorts.

	Robotic‐assisted TKA (*n* = 46)		Conventional TKA (*n* = 41)		*p* Value*
	*N*	(%)	*N*	(%)	
9 mm	38	(83)	28	(68.3)	0.122
11 mm	8	(17)	9	(22.0)	0.597
13 mm	0	(0)	4	(9.8)	0.063

Abbreviations: mm, millimetres; TKA, total knee arthroplasty.

* Statistically significant with *p* < 0.05.

### Clinical and radiological outcomes

Postoperative ROM at 2 months was similar across both groups, as was the KSS at the final follow‐up. The KSS Function score at the final follow‐up was significantly higher in the conventional group (93, range 80–100 vs. 96, range 80–100; *p* = 0.048). However, there was no statistically significant difference in the change in KSS knee or KSS function scores between the two groups before and after surgery. Postoperative HKA measurements were comparable in both cohorts: mean 179° ± 2° (range 173°–183°) in the robotic group and 177° ± 17° (range 171°–188°) in the conventional group (Table 3).

**Table 3:** Overview of the postoperative clinical and radiological outcomes at 2 months.

Variable	Robotic‐assisted TKA (*n* = 46)		Conventional TKA (*n* = 41)		*p* Value*
	Mean ± SD	(Min–Max)	Mean ± SD	(Min–Max)	
Flexion contracture (degrees)	1.2 ± 2.3	(0.0–10.0)	0.6 ± 1.5	(0.0–5.0)	0.281
Flexion (degrees)	111.5 ± 13.7	(60.0–130.0)	115.0 ± 13.3	(85.0–135.0)	0.232
KSS Knee	91.9 ± 7.5	(59.0–100.0)	93.5 ± 5.4	(80.0–100.0)	0.276
∆ KSS Knee	28.5 ± 13.4	(6.0–62.0)	33.4 ± 15.9	(5.0–70.0)	0.272
KSS Function	92.5 ± 6.9	(80.0–100.0)	95.5 ± 6.9	(80.0–100.0)	0.048*
∆ KSS Function	24.6 ± 13.2	(0.0–65.0)	28.9±15.6	(0.0–75.0)	0.142
HKA angle (degrees)	179.4 ± 1.9	(173.0–183.0)	176.8 ± 1.7	(171.0–188.0)	0.311
Delta HKA (degrees)	−1.0 ± 4.1	(−10.0–7.0)	0.3 ± 4.3	(−10.0–9.0)	0.141
Last follow‐up (month)	16.1 ± 8.1	(12.0–37.0)	30.8 ± 14.6	(12.0–62.0)	<0.0001*

Abbreviations: HKA, hip–knee–ankle angle; KSS, Knee Society Score; SD, standard deviation; TKA, total knee arthroplasty; ∆, the pre–post difference in KSS (postoperative − preoperative).

* Statistically significant with *p* < 0.05.

The rate of early complications at 2 months was similar in both groups (*p* = 0.34). The robotic‐assisted group reported three complications (6.5%): one case of scar revision surgery due to early dehiscence; one case of arthrofibrosis requiring mobilization under general anaesthesia and one case of early‐onset periprosthetic joint infection (PJI), which was treated with debridement, antibiotics and implant retention (DAIR). The conventional TKA group reported one complication (2.4%): a TKA revision due to chronic PJI.

## DISCUSSION

The most significant finding of our study was that operative time did not increase when robotic assistance was used for post‐HTO TKA compared to the conventional technique. Additionally, no clinically meaningful differences were observed in ROM at 2 months or KSS at final follow‐up between the two groups. There were differences between the groups regarding implant constraint, with a lower proportion of constrained implants in the robotic‐assisted group. Similarly, additional procedures such as tibial tubercle osteotomy or extensive lateral release were more frequently performed on patients in the conventional TKA group. There were no significant differences observed between the two groups regarding early complication rates or postoperative HKA angle.

Both the robotic‐assisted and conventional TKA cohorts in this study reported excellent KSS scores postoperatively. While there was a statistically significant, but clinically not meaningful, difference in the postoperative KSS Function score between both groups, it may have been caused by the more advanced osteoarthritis (as indicated by higher Ahlbäck scores) in the conventional cohort, possibly favouring their possible postoperative improvement of the KSS. As well, the conventional group also had a higher number of prior HTOs performed using the closing wedge technique. According to Bastos et al., clinical, functional and radiological outcomes were similar in patients who underwent conventional TKA following HTO, regardless of whether the procedure involved an opening or closing wedge technique [4].

Furthermore, our study found that integrating robotic assistance into more complex cases, such as post‐HTO TKA, did not result in longer operative times than conventional TKA. These cases were beyond the learning curve of robotic‐assisted TKA; Zhang et al. [27] and Kayani et al. [14] both described a learning curve of seven out of eleven cases for achieving optimal operative times. While evidence on primary TKA without prior HTO suggests that robotic‐assisted TKA takes as long as, if not longer than, conventional TKA, this has not been reported for post‐HTO cases. The surgeon's experience in this study may have contributed to the neutral operative times observed in this patient cohort. Additionally, the ability to use and adapt to real‐time intraoperative data to fine‐tune bone resection and guide implant positioning may have reduced the complexity of post‐HTO TKA. This could have optimized knee kinematics while minimizing the need for additional soft tissue releases [13].

Interestingly, robotic‐assisted TKA was associated with the use of lower implant constraint in patients with prior HTO compared to the conventional technique. A higher proportion of CS inserts and a lower proportion of PS inserts were observed in the robotic‐assisted group, while the use of CCK inserts did not differ significantly between groups. Intraoperative planning that incorporates real‐time soft‐tissue assessment may explain these differences. However, given the baseline disparities between the two groups, no causal relationship can be established. Moreover, Favroul et al. [9] and Yacovelli et al. [26] found no differences in clinical, functional or radiological outcomes when comparing CS and PS TKA, despite the difference in implant constraint.

The level of implant constraint was also associated with differences in stem utilization between the two groups. Stemmed implants were used more frequently in the conventional group. In situations requiring greater constraint, stems can improve load distribution at the bone–implant interface, potentially reducing the risk of aseptic loosening. For instance, Moussa et al. found that there was an increased risk of aseptic loosening with stemless CCK implants [17]. Although stemmed implants might be beneficial in primary TKA for patients with coronal deformity (greater than ten degrees) [6], obesity [11] or with diminished bone quality [10], they also present several challenges. These include a higher risk of thigh or leg pain, the technical difficulty to prepare the intramedullary canal, increased costs and potentially a more difficult removal of the implant in case of revision surgery [18].

These findings should be interpreted with caution, as they may reflect underlying differences in patient characteristics, surgical indications or surgeon preference rather than a direct effect of the surgical technique.

This study has several limitations inherent in its retrospective design. These include the potential for selection bias and the inability to establish causal relationships. It should also be acknowledged that radiological parameters are subject to intra‐ and interobserver variability, and that no reproducibility analysis was conducted. The relatively short follow‐up period restricts the evaluation of long‐term outcomes. However, patient‐reported outcome measures tend to remain consistent within the first 1–2 years after surgery, which could offset this issue [21].

Baseline differences between the robotic‐assisted and conventional TKA cohorts, including the higher Alhbäck score, ASA score and prevalence of prior closing wedge HTO in the conventional group, may have influenced the results. In this context. It is difficult to determine the extent to which these differences impacted the need for additional procedures, such as tibial tubercle osteotomy or lateral release.

Finally, the decision to use increased implant constraint or stemmed components was left to the discretion of the operating surgeon and may have been influenced by multiple factors, including intraoperative assessment, bone quality and resection accuracy.

## CONCLUSION

Robotic‐assisted TKA was associated with similar operative time and comparable short‐term clinical and functional outcomes to conventional TKA in patients with post‐HTO osteoarthritis. It was also associated with a reduced need for additional procedures, stemmed components and constrained implants. These findings should be interpreted with caution, given baseline differences between groups. Further research is required to evaluate long‐term implant survivorship and functional outcomes.

## AUTHOR CONTRIBUTIONS

**Sarah Descombris**: Study design; data collection; statistical analysis; literature review and manuscript writing. **Guillaume Mesnard**: Study design; literature review and manuscript editing. **Hannes Vermue**: Literature review and manuscript writing. **Cécile Batailler**: Literature review and manuscript editing. **Elvire Servien**: Literature review and manuscript editing. **Sébastien Lustig**: Study design; supervision; literature review and manuscript editing. All authors read and approved the final manuscript.

## CONFLICT OF INTEREST STATEMENT

C. B. is a consultant for Stryker and Smith and Nephew. E. S. is a consultant for Smith and Nephew. S. L. is a consultant for Stryker, Heraeus; Institutional research support from Groupe Lepine, Amplitude; Editorial Board for Journal of Bone and Joint Surgery (Am). The remaining authors declare no conflict of interest.

## ETHICS STATEMENT

All procedures performed in studies involving human participants were in accordance with the ethical standards of the institutional and/or national research committee and with the 1964 Helsinki declaration and its later amendments or comparable ethical standards. Data collection and analysis were carried out in accordance with MR004 Reference Methodology from the Commission Nationale de l′Informatique et des Libertés (Ref. 2238218v0) obtained on 24 March 2025. The study was registered and filed on the Health Data Hub website. Informed consent was obtained from all individual participants included in the study.

## Data Availability

The data that support the findings of this study are available on request from the corresponding author. The data are not publicly available due to privacy or ethical restrictions.
